# Can cytokines response play a role in the treatment of fatal leptospirosis?

**DOI:** 10.22088/cjim.13.2.4

**Published:** 2022

**Authors:** Ahmad Alikhani, Kimia Mahmoodi, Leyla Delavaryan, Ebrahim Salehifar, Ali Reza Rafiei, Mahmoud Sadeghi Zavare

**Affiliations:** 1 Department of Infectious Diseases, Antimicrobial Resistance Research Center and Communicable Disease Institute, Mazandaran University of Medical Sciences, Sari, Iran; 2 Ghaemshahr Razi Hospital, Ghaemshahr, Iran; 3 Department of Clinical Pharmacy, School of Pharmacy, Mazandaran University of Medical Sciences, Sari, Iran; 4 Department of Immunology, School of Medicine, Mazandaran University of Medical Sciences, Sari, Iran; 5 Department of Infectious Diseases and Tropical Medicine, Research Center, Babol University of Medical Sciences, Babol, Iran

**Keywords:** Leptospirosis, Inflammatory cytokine, Treatment, Outcome, IL10

## Abstract

**Background:**

The northern coastal regions of Iran are endemic for leptospirosis which may range from a subclinical illness to a progressively fatal disease. There has been growing evidence that inflammatory markers play a significant role in the severity and prognosis of leptospirosis. This study aimed to investigate inflammatory cytokines in patients with leptospirosis.

**Methods:**

This descriptive-analytical prospective study was performed in 75 patients over 18 years old who had a positive microscopic agglutination test (MAT) titer from January to June 2019. SPSS software Version 20 was used for statistical analysis and the significance level was considered as p<0.05.

**Results:**

The patients’ age enrolled in this study are from 21 to 75 years with a mean and standard deviation of 48.6±14.0. The male to female ratio in our participants was 54/21. Fever was the most common symptoms in 66 (88.0%) patients, followed by myalgia in 62 (82.7%) cases. The level of interleukin 10 was significantly higher in severe illness (P=0.003) and fatal cases (p<0.028) compared with recovered patients. The level of TNF-α level was also higher in the severe illness and Weil's syndrome compared with the mild kind (P=0.022).

**Conclusion:**

Our results showed that the levels of TNF-α and IL-10 significantly increased in severe leptospirosis. Also, IL-10 was significantly higher in fatal cases. The inhibition of IL-10 production might play an important role in decreasing the risk of fatal outcomes in leptospirosis.

Leptospirosis is a world-wide zoonotic disease which spread from animals to humans, caused by bacteria Leptospira from the family of spirochetes (1). The most common sources of infection are contact with the urine of infected animals and/or contaminated soil or water. Rodents and domestic mammals, such as cattle, pigs and dogs, serve as major reservoir hosts (1, 2). Leptospira enter the blood stream via cuts, skin abrasions or mucous membranes. Leptospirosis is an occupational disease in developing countries such as Iran with the most prevalence in farmers, ranchers, slaughterhouse workers, butchers and fishermen during the hot seasons. In developed countries, leisure activities such as swimming, water skiing [delete] is the main source of the disease (1, 2). In the last few decades, leptospirosis has re-emerged globally and the prevalence is expected to rise due to the demographic shifts in favor of increased number of regions subject to urban flooding, climate change and worsening storms. The incidence and diagnosis of leptospirosis is significantly lower than expected due to the lack of knowledge about the disease and lack of available, rapid and accurate tests for the early diagnosis (3).

Non-specific manifestations of leptospirosis often lead to mis-diagnosis (4-6). In Iran, annually, many farmers refer to the medical centers with fever and chills, severe headaches, myalgia, nausea, vomiting, and conjunctival hemorrhage during the agricultural season. The undiagnosed leptospirosis leads to a variety of expensive and invasive tests (7). Direct contact with the microbial agents during an infection activates the innate immune system and generate the anti-inflammatory response by regulating the expression of cytokines which are believed to be involved in the pathogenesis of leptospirosis. Tumor Necrosis Factor alpha (TNF-α) is an acute phase reactant in early systemic inflammation which recruits leukocytes to the site of infection and/or tissue damage (8). The increase in plasma concentrations of TNF-α is associated with poor prognosis in patients with leptospirosis (9). Interleukin 10 (IL-10) is known as an anti-inflammatory cytokine with pleiotropic effects in regulating immunity and inflammation by regulating Th1 cytokine production (10).

Considering the role of the inflammatory cytokines in the development of severe leptospirosis, these markers may be beneficial for better diagnosis of the complicated cases of leptospirosis and determining further prognosis. The North of Iran, especially Mazandaran province, is an endemic region for leptospirosis. Considering the growing need for a faster method to identify severe forms of leptospirosis and its treatment, and regarding the limited available data on the role of the major pro-inflammatory markers, the present study aimed to investigate the role of acute phase serum cytokines including IL-6, IL-8, IL-10, IL-12 and TNF-α concentrations in the progression of leptospirosis.

## Method

This descriptive-analytical study was performed on 75 patients with over 18 years of age who referred to Razi Hospital, Ghaemshahr, Iran, between April to June 2019, with laboratory-confirmed leptospirosis. Razi Hospital is an educational hospital affiliated to Mazandaran University of Medical Sciences which serves as a referral infectious diseases center in the North of Iran. Annually, among the more than 100 leptospirosis 100 admitted in this hospital considering its excluding criteria, 75 patients are included ultimately.

The diagnosis of disease was confirmed using the microscopic agglutination test (MAT), or ELISA (NovaTec Immundiagnostica GmbH, Germany, IgM & IgG). The positive MAT criteria included at least a fourfold rise in antibody titers between acute and convalescent sera after 14-30 days or a single titer of ≥ 400 in one or more samples. We collected 5 ml of blood from each participant with mild and severe leptospirosis and Weil’s syndrome. The whole blood samples were centrifuged. 

The isolated serum was stored at -20 °C and tested to measure the level of cytokines. Serum levels of IL-6, IL-8, IL-10, IL-12, and TNF-α were measured by the quantitative sandwich enzyme immunoassay using appropriate ELISA kits (R&D, CA). Briefly, the plates were coated by the desired anti-human antibody as the capture antibody for 24 hours at 4°C. Subsequently, 100 μl of standards or sera were added and the procedure was performed according to the manufacturer's instructions. Reference concentrations of the cytokines were used to prepare assay calibration. The absorption was determined with an ELISA reader (Biotek ELX800, USA) at 450 nm. The cytokine concentrations were interpolated from standard curves expressed in pg/ml. To avoid any bias, all samples were blindly analyzed without knowing the clinical status. 

Clinical features related to the disease progression and laboratory data were extracted by the interview and patients’ records, respectively. The inclusion criteria were patients over 18 years of age with possible diagnosis of leptospirosis due to the positive MAT titer and clinical features. Patients with other co-morbidities such as malignancies, autoimmune disorders (e.g., rheumatic diseases), and diabetes mellitus were excluded.

Statistical analysis was performed using the SPSS 20.0 (Statistical Package for Social Science, Version 20) statistical software package. Quantitative variables were described as mean and standard deviation (SD). The Kolmogorov-Smirnov test was used for the determination of quantitative data distribution. When the distribution of variables was normal, student’s t-test was used for the comparison of mean of 2 independent samples, and the non-parametric Mann-Whitney U test was used to compare non-normally distributed variables. Qualitative data was compared between the 2 groups using the chi-square (χ2) test. The difference was considered statistically significant when p<0.05.

The research protocol of this study was approved by the Research and Ethics Committee of Mazandaran University of Medical Sciences. (IR.MAZUMS.IMAMHOSPITAL.REC.1398.2824) written informed consent was obtained from all patients.

## Results

The patients’ age enrolled in this study ranged from 21 to 75 years with a mean and standard deviation of 48.6 ± 14.0 years. Most patients had 46 to 55 years and the male to female ratio in our participants was 54/21. Fever was the most common symptom in 66 (88%) patients, followed by myalgia in 62 (82.7%) cases. Among the 75 patients, the disease severity was mild in 39 (52.0 %), Severe in 31 (41.3%) and Weil Syndrome in 5 (6.7%) patients. The most common complications of leptospirosis were renal failure in 34 (45.3%) and hepatic involvement in 31 (41.3%) cases. 72 (96.0%) patients completely recovered, and 3 (4.0%) cases had fatal outcomes. Patients were divided into three groups based on the correction of platelet level: normal, reduced, and corrected with 29 (38.7%), 22 (29.3%) and 24 (32.0%) patients, respectively. The normal range regarding to up-to-date 2021 was considered as 150000 to 450000 / microl. The levels of IL-6, IL-8, IL-10, IL-12 and TNF-α at the presentation time were 3.4±3.4, 11.7±15.4, 17.1±17.6, 32.0±12.3 and 27.9±28.3, respectively. There was no statistically significant difference in the levels of cytokines between different age groups, genders, complications, response to treatment and outcome, (all P-values >0.05). 

Table 1 describes the level of inflammatory cytokines in patients with leptospirosis in both gender groups.

The level of IL-10 was significantly higher in patients with Weil’s syndrome (23.41±19.78) compared with mild (5.02±6.13) and severe (11.15±12.43) forms of the disease (P=0.003). Also, the level of TNF-α was significantly higher in patients with Weil’s syndrome compared with mild and severe forms (36.44±31.22 vs 14.59±12.79 and 19.36±22.84, respectively,( P=0.022). There was no significant difference between the level of IL-6, IL-8 and IL-12 with different severities of the leptospirosis (table 2).

IL-10 was significantly higher in patients with fatal disease (P=0.028). All three patients died of alveolar hemorrhage. There was no statistically significant difference for other cytokines regarding the mortality rate (table 3). The level of IL-6 was higher in patients with reduced platelet correction (4.86±5.32), compared with normal (2.80±1.62) and corrected groups (2.68±1.47), (P=0.038). The level of IL-10 was significantly higher in patients with normal platelet correction (22.43±19.88) compared with other two groups (P=0.023). The level of TNF-α‌ was 40.30±32.24, 25.99±25.38, and 13.73±17.51 in normal, decreased and corrected platelet, which showed a statistically significant difference (P=0.003). In other inflammatory cytokines including IL-8 and IL-12, based on different platelet correction, no statistically significant difference was observed (p>0.05). Data are shown in table 4.

**Table 1:** Levels of inflammatory cytokines in patients with leptospirosis by sex

**Variables**	**Sex** **Female**	**Male**	**Pvalue**
IL-6	33± 2.2	3.5± 3.7	0.852
IL-8	9.2±13.5	12.7±16.1	0.386
IL-10	17.3±18.8	17.1±17.4	0.961
IL-12	37.1±12.7	30.0±11.6	0.053
TNF-a	28.4±29.5	27.7±28.1	0.930

**Table 2:** Levels of inflammatory cytokines in patients with leptospirosis based on disease Severity

**Variable**	**Disease Severity** **Mild Severe**		**Weil’s syndrome**	**pvalue**
IL-6	2.5±1.01	3.39±4.74	3.57±0.03	0.806
IL-8	8.36±6.88	10.93±14.59	12.74±16.88	0.786
IL-10	5.02±6.13	11.15±12.43	23.41±19.78	0.003
IL-12	32.02±12.73	32.86±11.83	26.06±11.96	0.521
TNF	14.59±12.79	19.36±22.84	36.44±31.22	0.022

**Table 3:** Levels of inflammatory cytokines in patients with leptospirosis by mortality

**variable**	**Mortality** **No yes**		**pvalue**
IL-6	3.5±3.4	2.4±0.5	0.601
IL-8	11.3±15.2	20.4±20.6	0.324
IL-10	1.9±1.1	17.8±17.7	0.028
IL-12	32. 2±12.0	27.3±20.0	0.508
TNF	27.7±28.4	32.2±31.7	0.792

**Table 4:** Levels of inflammatory cytokines in patients with leptospirosis based on correction

**Variable**	**Normal**	**Decreased**	**Corrected**	**pvalue**
IL-6	2.80±1.62	4.86±5.32	2.68±1.47	0.038
IL-8	12.61±20.06	12.73±13.27	9.38±9.84	0.707
IL-10	22.43±19.88	18.13±18.26	9.01±9.78	0.023
IL-12	33. 65±12.29	31.28±12.02	30.52±12.78	0.635
TNF-a	40.30±32.24	25.99±25.38	13.73±17.51	0.003

## Discussion

The main purpose of this study was to determine cytokines response in severe form and fatal leptospirosis. IL-10 and TNF-α level were significantly higher in severe form and Weil’s syndrome. In addition, IL-10 was significantly higher in fatal cases, all of which were after intra-alveolar hemorrhage. Reis. et al. (11) confirmed that an increase in IL-10 and TNF-α was associated with higher disease severity. Progression from a non-specific immune response to an acquired immune response with Th2 predominance, including inhibition of Th1 with excessive production of IL-10, plays an important immune-pathogenic role in estimating the severity and mortality rate of leptospirosis. This finding is consistent with the results of studies in animal models of leptospirosis, which have shown that increased production of IL-10 is associated with increased mortality (7). In addition, previous studies in leptospirosis have shown a significant association between IL-10 and fatal outcomes (12). 

Studies have shown that components of the leptospira bacterium, including glycoproteins and lipopolysaccharides, are released after bacterial lysis, which inhibit Na / K-ATPase enzyme and could cause direct tissue damage or increased production of plasma non-esterified fatty acid (NEFA) that result in excessive release of inflammatory mediators. These factors lead to a severe immune response with multiple organ damage, which are seen in the severe forms of the leptospirosis (13). In the previous studies, higher levels of interleukin 2, 4, 6, 8, 10, and 17, as well as TNF-α, were significantly associated with the disease severity. In addition, patients with severe illness or fatal cases had higher levels of IL-6 and 8 (11). A previous study in Thailand have shown that patients with organ involvement have significantly higher levels of IL-6, 8, and 10 in the acute phase serum (14). Chierakul et al. (15) showed that IL-6 and TNF-α were the most important pro-inflammatory cytokines in the serum of leptospirosis patients. Our results suggested determining the level of TNF-α in patient’s serum maybe as a favorable prognostic factor in leptospirosis patients.

In the present study, no significant relationship was found between the level of IL-6, and 8 with the severity and mortality rate of the leptospirosis. The evidence on this finding is not quite so clear. In a study by Mikulski et al. (16), which examined prognostic markers for the severity of leptospirosis, no significant relationship was found between severity and mortality with levels of IL-6, and 8. A similar result was obtained in a study by Tajiki et al.(17). In contrast, studies have shown that high levels of IL-6, and 8 were associated with the more severe forms and fatal cases of the disease (3, 18, 19). IL-8 is a pro-inflammatory mediator that induces chemotaxis of neutrophils and is associated with hepatitis caused by leptospirosis, whereas IL-6 disrupts the endothelial barrier and is associated with pulmonary hemorrhage syndrome (11, 20). One of the reasons for these controversial results could be the timing of IL-6, and 8 sampling and the different severity of the disease in patients. The rate of these cytokines in the present study was higher in patients with severe forms of the disease compared with patients with mild form; however, this difference was not statistically significant. Also, in the present study, platelet correction was associated with decreased levels of IL-6, TNF-α, and IL-10. The use of anti-IL-10 therapies should be considered in the prevention of fatal outcomes. However, due to the lack of enough studies in this field, more studies are needed to target IL-10 in the treatment of the severe forms of the leptospirosis. One of the limitations of this study was the small sample size, which can make it difficult to identify significant relationships in the whole community. However, an attempt was made to increase the accuracy of the study as much as possible by examining multiple inflammatory cytokines and the accurate diagnosis of [delete] leptospirosis with MAT test. In conclusion, the results show that TNF-α and IL-10 significantly increased in severe leptospirosis and IL-10 is significantly higher in fatal cases. Platelet correction is also associated with decreased levels of IL-6, TNF-α, and IL-10. To balance between TNF- α and IL-10 production might play an important role in decreasing the risk of death from leptospirosis. This may be provided by anti TNF- α or IL-10 products in these patients.

**Recommendations: **The use of specific anti-inflammatory cytokine products such as IL-10 will shed light on the treatment and better prognosis of leptospirosis patients [delete]. Our results suggest determining and balancing [delete] the level of TNF-α and IL-10 in patient’s serum as useful prognostic factors in leptospirosis patients.
